# Repeatability, Reproducibility and Agreement of Central Corneal Thickness Measurements by Two Noncontact Pachymetry Devices

**Published:** 2019

**Authors:** Ozkan KOCAMIS, Rasit KILIC

**Affiliations:** 1 Ahi Evran University Medical Faculty, Department of Ophthalmology, Kirsehir, Turkey

**Keywords:** TRK-2P, Lenstar 900, Central Corneal Thickness, Repeatability, Reproducibility

## Abstract

This study was designed to assess the repeatability, reproducibility, and agreement of Noncontact Pachymetry (NPC) (Topcan TRK-2P) and the non-contact optical low coherence reflectometer (Lenstar LS 900) devices for measuring Central Corneal Thickness (CCT) of healthy corneas. A total of 82 healthy volunteers were evaluated. The first observer used both the TopconTRK-2P and Lenstar 900 devices while the second observer only used the TopconTRK-2P for the measurements. The measurements with either device were repeated three times for each patient, consecutively. The central corneal thickness measurements with the Topcon TRK-2P revealed mean ± Standard Deviation (SD) values of 553.1 ± 36.1 micrometer (µm) for the first observer and 552.3 ± 35.9µm for the second observer and the mean ± SD of CCT was 537.3 ± 34.8µm with the Lenstar 900. The difference between the CCT measurements of the observers using the Topcon TRK-2P (P = 0.142) was insignificant. However, significantly lower measurements were found with the Lenstar 900 compared with the Topcon TRK-2P (P ˂ 0.001). The central corneal thickness measurements obtained by the Topcon TRK-2P were found to have high repeatability for both observers with a lower SD, less than 1% Coefficient of Variation (CV) and higher than 0.99 Intra-Class Correlation Coefficient (ICC) (Observer 1: 3.77 SD, 0.68 CV and 0.995 ICC; the second observer: 3.58 SD, 0.65 CV and 0.995 ICC). There was an excellent inter-observer reproducibility between the two observers for Topcon TRK-2P with 2.71 SD, 0.49 CV, and 0.994 ICC. The Bland-Altman plot showed high agreement between the two devices. These results suggest that the TopconTRK-2P is a reliable device for evaluating CCT in healthy corneas compared with Lenstar 900.

## INTRODUCTION

Accurate Central Corneal Thickness (CCT) measurements can be useful for determining endothelial cell function, refractive surgery screening and planning, and finding out the true Intraocular Pressure (IOP) [1-4]. The most important predictor of ocular hypertension development to glaucoma is CCT. According to the results of the Ocular Hypertension Treatment study, a person whose CCT is 40 micrometers (μm) thinner than the normal mean, has a 71% greater chance of developing glaucoma [5]. The results of the meta-analysis of Doughty and Zaman [4] showed that 10% change in the CCT would alter the IOP by 4.3 millimeter of mercury (mmHg). Central corneal thickness also plays an important role in the selection of ablation amount and diameter and the laser surgical method to be employed in refractive surgery [6]. The central corneal thickness is very useful in the diagnosis of corneal diseases, such as Fuchs’ corneal dystrophy and keratoconus [7]. The pachymetry measurements must be prompt, precise, and reproducible to be considered as a screening protocol by the eye care practitioners [8]. To measure CCT, a wide range of already-in-use and advanced devices include conventional Ultrasound Pachymetry (UP), confocal biomicroscopy, Scheimpflug imaging, Optical Coherence Tomography (OCT), and Optical Low-Coherence Reflectometry (OLCR) [9-14]. 

The Topcon TRK-2P automated optical pachymeter (Topcon, Tokyo, Japan) is a modern instrument and currently mingles corneal pachymetry with other screening issues, such as non-contact IOP measurement, auto refraction, and keratometry. The Lenstar 900 device (Haag-Streit AG, Köniz, Switzerland) uses the OLCR system and has been used to measure CCT, and to obtain other optical data, such as anterior chamber depth, lens thickness and axial length, and to perform keratometry and pupillometry [12-14].

Accurate measurements of CCT are of critical importance when evaluating many ocular disorders. A new device or technique should therefore be compared with others, such as UP and OLCR, so that its accuracy is known [15, 16]. The aim of this observational study was to compare CCT measurements between the Topcon TRK-2P and Lenstar 900 devices. The intra-observer repeatability, inter-observer reproducibility and the level of agreement between the two devices were evaluated.

## METHODS

The study was conducted on 82 volunteers at the Ahi Evran University Training and Research Hospital's Ophthalmology Clinic from June 2018 to July 2018. The study conformed to the principles of the Helsinki Declaration. The ethics committee consent was approved from the Ahi Evran University Faculty of Medicine's Clinical Studies Ethics Committee. Informed consent was obtained from each case, who received a comprehensive explanation about all procedures involved in the study.

The study excluded patients with past corneal surgery, corneal disorder or disease, ocular disease, such as glaucoma, systemic disorder, such as hypertension or diabetes mellitus, those using topical or systemic drugs, patients with a refractive error that was larger than 5 Diopter (D) sphere or 3D cylinder, and patients using contact lenses.

A medical history was obtained from all volunteers and the results of the ophthalmic eye examination were recorded. The examination included determination of the Corrected Distance Visual Acuity (CDVA), anterior segment examination using slit-lamp bio-microscopy, dilated-pupil fundus examination, and IOP measurement with an air puff-tonometer (Topcon, Tokyo, Japan). The right eye was evaluated in all volunteers of this study.

The central corneal thickness of all volunteers was measured randomly with the TopconTRK-2P (Topcon, Tokyo, Japan) automated optical pachymeter or the Lenstar 900 (Haag-Streit AG, Köniz,Switzerland) OLCR instrument. The first observer made measurements with both the TopconTRK-2P and the Lenstar 900 instrument whereas the second one only used the TopconTRK-2P. The measurements with either device were repeated three times for each patient. The patient was asked to blink and wait for at least 30 seconds to maintain the continuity of the tear film during the measurements.

The SPSS version 20.0 software was used for statistical evaluation. Descriptive statistical methods were used to evaluate data regarding age, gender, IOP, and Spherical Equivalent (SE). The paired samples *t* test was used to compare the CCT measurements between observers, using the Topcon TRK-2P, and also between the devices. Intra-observer repeatability was assessed by calculating the mean of the Standard Deviations (SDs), the Coefficient of Variation (CV), and the Intra-class Correlation Coefficient (ICC). Inter-observer reproducibility was evaluated by analyzing the ICC. The Bland-Altmanplot test was used to determine the compliance between the measurements of the two observers [17]. The intra-class correlation coefficient is accepted to indicate a weak correlation when the value is 0.75 or below, moderate when 0.75 to 0.90 and high when 0.90 and above [18]. Low SD and CV values indicate good repeatability.

## RESULTS

**Figure 1:**
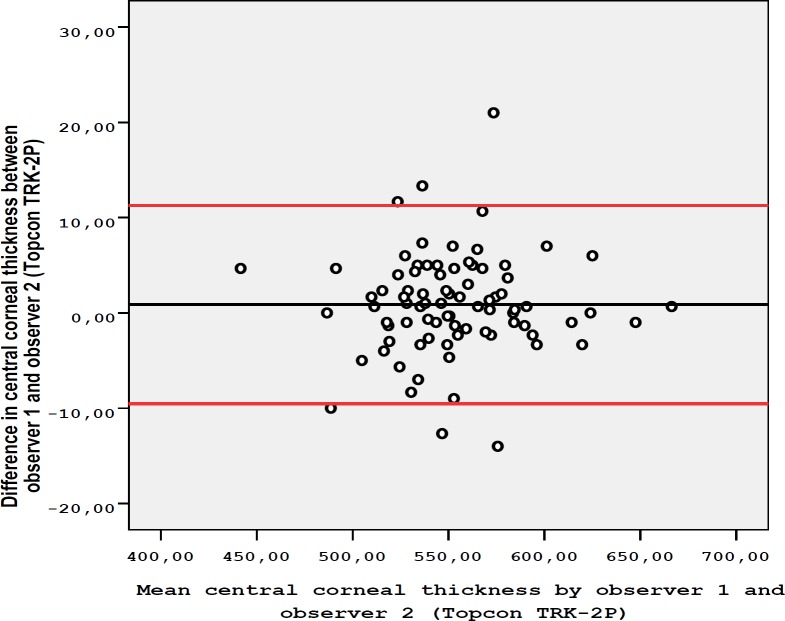
Bland-Altman Plot shows the Differences in Central Corneal Thickness Measurements between Observer1 and Observer 2 using the Topcon TRK-2P. The Black Line Represents the Mean Difference and the Red Lines represent the 95% Confidence Interval (CI) Limits of Agreement.

**Figure 2:**
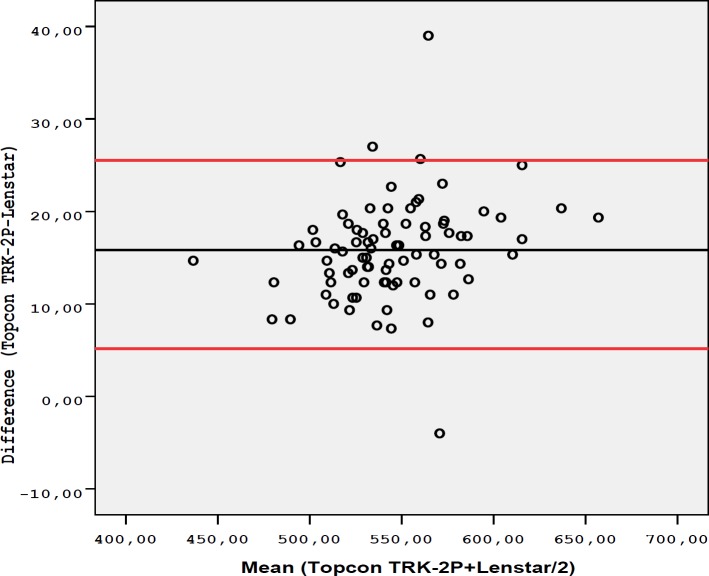
Bland-Altman Plot indicates the Differences in Central Corneal Thickness Measurements obtained by Observer 1 using the Topcon TRK-2P and Lenstar 900. The Black Line represents the Mean Difference and the Red Lines represent the 95% Confidence Interval (CI) Limits of Agreeme.

The age of the 82 cases included in the study was 40 ± 9.9 years (mean ± SD). The gender distribution was 45 (54.9%) females and 37 (45.1%) males. The mean ± SD of IOP was 14.5 ± 2.8 mmHg and the mean ± SD of SE was -0.59 ± 1.26 D. The mean CCT measurements with the Topcon TRK-2P were 553.1 ± 36.1 µm for the first observer and 552.3 ± 35.9 µm for the second observer. The mean ± SD of CCT with the Lenstar 900 was 537.3 ± 34.8 µm. The difference between the CCT measurements of the observers, using the Topcon TRK-2P (P = 0.142), was insignificant. However, significantly lower measurements were found with the Lenstar 900 compared to the Topcon TRK-2P (P ˂ 0.001). When the intra-observer repeatability was evaluated, CCT measurements obtained by Topcon TRK-2P were found to have high repeatability for both observers, with a lower SD, CV less than 1%, and ICC higher than 0.99 (Table 1). Regarding intra-observer repeatability for the first observer, CCT measurements obtained by Lenstar 900 were also found to have excellent repeatability with SD value of 1.25 SD, CV of 0.23%, and ICC of 0.999. These results indicated excellent intra-observer repeatability for both devices. When inter-observer reproducibility between the two observers was assessed for Topcon TRK-2P, there was an excellent agreement (Table 1 and Fig 1). The Bland-Altmanplot in Fig 1 shows high agreement between the two observers when using Topcon TRK-2P. The Bland-Altman plot also showed high agreement between the two devices (Fig 2). Table 2 indicates differences in CCT measurements between the observers when using Topcon TRK-2P and between the two devices.

**Table 1:** Repeatability and Reproducibility of Central Corneal Thickness (CCT) using the Topcon TRK-2P

	Mean ± SD CCT	SD	CV (%)	ICC (95% CI)
Repeatability for observer1	553.1 ± 36.1 µm	3.77	0.68	0.995 (0.993-0.996)
Repeatability for observer2	552.3 ± 35.9 µm	3.58	0.65	0.995 (0.993-0.997)
Reproducibility betweenObserver1 and Observer2	552.7 ± 35.9 µm	2.71	0.49	0.994 (0.991-0.996)

CI: confidence interval; µm: micrometer; CV: coefficient of variation of repeated measurements; ICC: intraclass correlation coefficient; SD: Standard Deviation; %: percentage.

**Table 2:** Bland-Altman Plot lower and upper Limits of Agreement between Observers using by Topcon TRK-2P and between Devices

Comparison	Difference	Limits of agreement	
	** Mean ± SD **	**Lower**	**Upper**
Observer 1 vs. Observer 2 (Topcon TRK-2P)	0.87 ± 5.31 µm	-9.54	11.28
Topcon TRK-2P vs. Lenstar 900	14.96 ± 4.01µm	7.10	22.82

SD: Standard Deviation; µm: micrometer; vs: versus.

## DISCUSSION

This study used TRK-2P that is a higher version of TRK1P in this study. While the difference between the CCT measurements of the observers using the Topcon TRK-2P was insignificant, significantly lower measurements were found when Lenstar 900 was compared with Topcon TRK-2. Regarding intra-observer repeatability for the first observer, CCT measurements obtained with Lenstar 900 were also found to have excellent repeatability with an SD value of 1.25, CV of 0.23%, and ICC of 0.999. These results showed excellent intra-observer repeatability for both devices. When inter-observer reproducibility between two observers using Topcon TRK-2P was assessed, there was an excellent agreement. 

The Ultrasonic Pachymetry (UP) is the gold standard for evaluating CCT in daily practice. However, topical anesthesia use and the contact method are the limitations of this technique. Devices using non-contact methods, including optical coherence tomography, optical, low-coherence reflectometry, corneal topography techniques, and noncontact specular microscopy have now been developed to measure CCT [19]. However, beside its accuracy, repeatability, and reproducibility of the measuring instrument are very important for a device to be introduced in clinical practice. Many studies have been carried out to assess the repeatability, reproducibility, and accuracy of new devices in the literature. The Lenstar 900 device has already been shown to have a high repeatability and high inter-observer reproducibility, which is comparable to USP [20-24] . 

The reliability of Lenstar 900 has been compared with other devices in the literature [15, 16, 21]. The highest correlation between Lenstar 900 and UP was found by Borrego-Sanz et al. [22]. Similarly, another study by Tai et al. on 184 eyes showed a high correlation between Lenstar 900 and UP [23]. In their retrospective study on 50 patients, Beutelspacher et al. [18] found a high degree of correlation (97%) and agreement (r = 0.929) between the OLCR and UP in the measurement of CCT. Bayhan et al. [24] assessed the repeatability of measurements between the Scheimpflug-Placido Topographer, OLCR, SD-OCT (RTVue), and UP. They demonstrated that all devices had high and comparable repeatability in a healthy population. It is therefore well established that Lenstar 900 is a reliable device. The researchers therefore compared TopconTRK-2P with Lenstar 900 in this study.

The first study with a combination of tonometry and pachymetry was performed by Schiano et al. [25]. In their study, CCT measurements with the Tonopachy NT-530P were found to be 13 μm lower than ultrasonic pachymetry and 3.7 μm higher than a slit scanner laser (Orbscan). The current study found CCT, with the TopconTRK-2P device, to be approximately 15 µm higher in a statistically significant manner compared to the Lenstar 900. The TRK1P, the previous version of TopconTRK-2P, has also been compared with OCT and US pachymetry in the literature. Wells et al. [26] demonstrated that CCT measurements with the Topcon TRK-1P and OCT were significantly lower than with UP, with a difference of approximately 30µm and 17µm, respectively, and a Pearson correlation coefficient of 0.96 (P < 0.001) and 0.98 (P < 0.001), respectively. The TRK-1P measurement of CCT was lower than the value obtained by ultrasound. They explained this difference with the local anesthetic swelling effects on the cornea. The current study found CCT to be about 15 µm thicker with TRK-2P compared to Lenstar 900. In general, OLCR measurements were slightly lower than with UP [26, 27]. 

The present study only evaluated normal corneas of healthy subjects. Therefore data are not available regarding the agreement between TRK-2P and other methods when measuring compromised corneas secondary to pathological alterations or surgical interventions. Further studies are needed to evaluate the reliability of theTRK-2P in measuring CCT in pathological corneas.

## CONCLUSION

In conclusion, this study found a CCT value that was 15µm thicker with the Topcon TRK-2P compared to the Lenstar 900. There was perfect intra-observer repeatability and inter-observer reproducibility with the TopconTRK-2P. There was also an excellent agreement between the TopconTRK-2P and Lenstar 900. These results suggest that TopconTRK-2P is a reliable device for evaluating CCT in healthy corneas.
